# Probucol Reduces Testicular Torsion/Detorsion-Induced Ischemia/Reperfusion Injury in Rats

**DOI:** 10.1155/2017/5424097

**Published:** 2017-09-10

**Authors:** Si-Ming Wei, Yu-Min Huang, Jian Zhou

**Affiliations:** ^1^Department of Surgery, School of Nursing, Zhejiang Chinese Medical University, Hangzhou City, Zhejiang Province 310053, China; ^2^Department of Urology, Third Affiliated Hospital of Hangzhou City, Zhejiang Chinese Medical University, Hangzhou City, Zhejiang Province 310009, China; ^3^Department of Clinical Medicine, Hangzhou Medical College, Hangzhou City, Zhejiang Province 310053, China; ^4^Department of Surgery, First Affiliated Hospital, Zhejiang Chinese Medical University, Hangzhou City, Zhejiang Province 310006, China

## Abstract

This study investigated the effect of probucol, a potent antioxidant, on testicular torsion/detorsion-induced ischemia/reperfusion injury attributable to excess reactive oxygen species released by neutrophils. Sixty male Sprague-Dawley rats were randomly divided into sham-operated control, ischemia-reperfusion, and probucol-treated groups. In the ischemia-reperfusion group, testicular detorsion was performed after 2 hours of left testicular torsion. In the probucol-treated group, after performing the same surgical procedures as in the ischemia-reperfusion group, probucol was given intraperitoneally at testicular detorsion. Orchiectomy was performed to evaluate protein expression of E-selectin which is an endothelial cell adhesion molecule and mediates neutrophil adhesion to vascular endothelium, myeloperoxidase activity (a mark of neutrophil accumulation in the testis), malondialdehyde level (an indicator of reactive oxygen species), and spermatogenesis. E-selectin protein expression, myeloperoxidase activity, and malondialdehyde level were significantly increased, and testicular spermatogenesis was significantly decreased in the ipsilateral testes in the ischemia-reperfusion group, compared with the control group. The probucol-treated group showed significant decreases in E-selectin protein expression, myeloperoxidase activity, and malondialdehyde level and significant increase in testicular spermatogenesis in the ipsilateral testes, compared with the ischemia-reperfusion group. These findings indicate that probucol can protect testicular spermatogenesis by reducing overgeneration of reactive oxygen species by inhibiting E-selectin protein expression and neutrophil accumulation in the testis.

## 1. Introduction

Testicular torsion is a common urologic emergency that usually affects male newborns, children, and adolescents. It results from a rotation of the spermatic cord and leads to compromised testicular blood flow. Rapid diagnosis and surgical detorsion are essential to restore blood flow to the testis and avoid testicular necrosis. Despite return of blood flow after testicular detorsion, testicular atrophy is a common outcome [1]. The primary pathophysiology of testicular torsion and detorsion is testicular ischemia-reperfusion injury. Abundant amounts of reactive oxygen species are produced during ischemia-reperfusion [2]. These reactive oxygen species, including superoxide anions, hydrogen peroxide, nitric oxide, and hypochlorous acid, can lead to loss of cell viability by causing lipid peroxidation in the cellular membrane, protein denaturation, and DNA damage [3]. Mammalian testes are highly sensitive to reactive oxygen species damage and particularly to lipid peroxidation due to testicular high content of polyunsaturated fatty acids [4]. We have reported that curcumin [5] and rutin [6] can protect against testicular torsion/detorsion-induced ischemia/reperfusion injury in rats by reducing reactive oxygen species.

Probucol is a clinically used lipid-lowering drug with potent antioxidant and anti-inflammatory properties [7]. Early studies have demonstrated that probucol can attenuate ischemia-reperfusion injury in the kidney, heart, and brain [8–10]. However, the effect of probucol on testicular ischemia-reperfusion injury has not been evaluated previously. Our purpose in this study was to investigate whether probucol can protect the testis from ischemia-reperfusion injury in rats.

## 2. Materials and Methods

### 2.1. Animals

Sixty male Sprague-Dawley rats (8 weeks old) weighing between 250 and 300 g were obtained from Shanghai Laboratory Animal Center (Shanghai City, China). They were maintained on a 12-hour light/dark cycle at room temperature (21°C ± 1°C) and humidity (55% ± 5%) and were provided with standard rodent food and water ad libitum. This experimental study was performed with the approval of the ethics committee on animal research at our university, in compliance with the principles of experimental animal care and use as published by the US National Institutes of Health.

### 2.2. Drugs and Reagents

Probucol, anti-*β*-actin antibody, hexadecyltrimethylammonium bromide, *o*-dianisidine dihydrochloride, and hematoxylin and eosin were purchased from Sigma Chemical Company (St. Louis, MO, USA). Protein assay kit was obtained from Bio-Rad Laboratories (Hercules, CA, USA). The anti-E-selectin antibody, horseradish peroxidase-linked secondary antibody, and enhanced chemiluminescence reagent were purchased from Santa Cruz Biotechnology (Santa Cruz, CA, USA). Malondialdehyde assay kit was obtained from Nanjing Jiancheng Institute of Bioengineering (Nanjing City, China). All other reagents were of the highest grade commercially available.

### 2.3. Experimental Procedure

Sixty male Sprague-Dawley rats were randomly assigned into three groups as follows: control group (*n* = 20), ischemia-reperfusion group (*n* = 20), and probucol-treated group (*n* = 20). They were anesthetized with 50 mg/kg ketamine injected intraperitoneally. All surgical procedures were done by the same surgeon under sterile conditions. A left-sided ilioinguinal incision was performed to access the left testis. In the control group, the left testis was taken out through the incision and an 11-0 atraumatic silk suture was placed through the tunica albuginea. The left testis was returned to the scrotum, and the incision was closed using a 4-0 silk suture. In the ischemia-reperfusion group, testicular ischemia was created by 720° rotation of the left testis in a counterclockwise direction. The left testis was fixed to the scrotal wall with an 11-0 atraumatic silk suture. After 2 hours of ischemia, the fixing suture was removed, and the left testis was released in a clockwise direction to initiate reperfusion. The testis showed gross improvement in parenchymal blood flow and was relocated into the scrotum. For the probucol-treated group, in addition to the same surgical procedures carried out in the ischemia-reperfusion group, 300 mg/kg of probucol was administered intraperitoneally to each rat at reperfusion. Some other studies have shown that probucol at a dose of 300 mg/kg is effective in the treatment of ischemia-reperfusion injury in rat heart [9] and kidney [8]. As a result, this dose was chosen in our rat testicular ischemia-reperfusion injury model. Four hours after reperfusion, half of the rats in each group were sacrificed and the testes were harvested for evaluation of E-selectin protein expression, myeloperoxidase activity, and malondialdehyde level. The rest of the rats in each group were euthanized 3 months after reperfusion, and the testes were removed for investigation of testicular spermatogenesis.

### 2.4. Determination of E-Selectin Protein Expression by Western Blot Analysis

Testicular tissues were homogenized in ice-cold lysis buffer (50 mM Tris HCl, pH 7.4, 5 *μ*g/ml aprotinin, 1 mM phenylmethylsulfonyl fluoride, 0.5% sodium deoxycholate, 150 mM NaCl, 0.5 mM ethylenediaminetetraacetic acid, 2 mM sodium orthovanadate, 0.1% sodium dodecyl sulfate, 1 mM dithiothreitol, 1% nonidet P-40, and 0.5 *μ*g/ml leupeptin). The homogenate was centrifuged at 4°C at 14,000*g* for 15 minutes, after which the supernatant was collected. Concentration of protein in the supernatant was quantified using the Bio-Rad protein assay kit. Protein sample (20 *μ*g) was boiled for 3 minutes and subjected to sodium dodecyl sulfate polyacrylamide gel electrophoresis. Separated protein was electrotransferred to nitrocellulose membrane. After blocking with 5% nonfat milk solution, the membrane was incubated with anti-E-selectin antibody or anti-*β*-actin antibody overnight at 4°C. Subsequently, the membrane was washed with Tris buffer and incubated with horseradish peroxidase-linked secondary antibody for 60 minutes at room temperature. Protein band on the membrane was visualized by an enhanced chemiluminescence reagent and autoradiography. Optical density of protein band was quantified using a GS-700 imaging densitometer (Bio-Rad Laboratories, Hercules, CA, USA). The densitometric ratio of the E-selectin band to the internal control *β*-actin band from the same sample indicated a relative expression level of E-selectin protein.

### 2.5. Measurement of Myeloperoxidase Activity

Testicular sample was homogenized in 50 mM potassium phosphate buffer and then centrifuged at 40,000*g* for 30 minutes. The supernatant of each sample was removed, and the pellet was suspended in 50 mM potassium phosphate buffer supplemented with 0.5% hexadecyltrimethylammonium bromide. The suspension was sonicated for 10 seconds, freeze-thawed 3 times, sonicated for 10 seconds, and centrifuged at 40,000*g* for 30 minutes. Supernatant was harvested, and a 100 *μ*l sample of supernatant was incubated with 0.0005% hydrogen peroxide and 0.167 mg/ml *o*-dianisidine dihydrochloride. The change in absorbance in each sample was detected at 460 nm using a spectrophotometer. One unit of myeloperoxidase activity is defined as the amount of enzyme which degrades 1 mM of peroxidase per minute. Myeloperoxidase activity was expressed as U/g tissue.

### 2.6. Malondialdehyde Analysis

The malondialdehyde levels in testicular samples were measured using the thiobarbituric acid reactive substance method described by Ohkawa and colleagues [11]. The values of malondialdehyde were expressed as nmol/mg protein.

### 2.7. Examination of Testicular Spermatogenesis

Testicular spermatogenesis was assessed by certain characteristics, including testicular weight, mean seminiferous tubular diameter, germ cell layer number, and mean testicular biopsy score. All testes were weighed and fixed in Bouin solution. Next, testicular tissues were dehydrated in an ethanol series and embedded in paraffin block. Section was cut into 5 *μ*m thicknesses and subjected to hematoxylin and eosin staining. The testicular section was examined in double-blinded manner under a light microscope by a single pathologist. Mean seminiferous tubular diameter, germ cell layer number, and mean testicular biopsy score were evaluated in 20 most circular tubules from each section. Light microscope with an eyepiece micrometer was used to measure mean seminiferous tubular diameter. We determined the germ cell layer number by counting the numbers of the germ cell layer from the basement membrane to the tubular lumen at 90°, 180°, 270°, and 360° and calculating the average value. The mean testicular biopsy score was performed using Johnsen's scoring system [12]. Under this scoring system, epithelial maturation in seminiferous tubule was scored from 1 to 10. A score of 10 represented complete spermatogenesis with many spermatozoa, regular germinal epithelium, and open tubular lumen. A score of 1 represented no cells in the seminiferous tubule.

### 2.8. Statistical Analysis

All statistical analyses were performed with computer software (Prism 4.0, GraphPad Software Inc., San Diego, CA, USA). Results were expressed as mean ± standard deviation. Differences in measured parameters among the groups were analysed by one-way analysis of variance. Student-Newman-Keuls test was used as a post hoc analysis for multiple comparisons. Student *t*-test was performed to compare data between ipsilateral and contralateral testes within the same group. Statistical significance was set at *P* value of less than 0.05.

## 3. Results

### 3.1. E-Selectin Protein Expression

**Figure 1:**
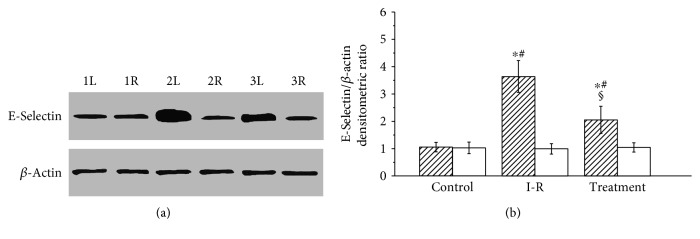
The protein expression level of E-selectin in testicular tissues. (a) Representative example of Western blot of E-selectin with *β*-actin as an internal control. Lanes 1L and 1R indicate the left (i.e., ipsilateral) and right (i.e., contralateral) testes in the control group. Lanes 2L and 2R indicate the ipsilateral and contralateral testes in the ischemia-reperfusion group. Lanes 3L and 3R indicate the ipsilateral and contralateral testes in the treatment group. (b) Densitometric ratio of the Western blot results in ipsilateral (hatched bars) and contralateral (open bars) testicular tissues in the control, ischemia-reperfusion (I-R), and treatment groups. The densitometric ratio of the E-selectin band to *β*-actin band from the same sample represents a relative expression level of E-selectin protein. Results are expressed as mean ± standard deviation; *n* = 10. ^∗^*P* < 0.05 versus the control group; ^#^*P* < 0.05 versus contralateral testes in the same group; ^§^*P* < 0.05 versus ipsilateral testes in the I-R group.

The testicular E-selectin protein expression levels in the control, ischemia-reperfusion, and probucol-treated groups are shown in Figure 1. Testicular ischemia-reperfusion caused a significant increase in E-selectin expression of the ipsilateral testes compared with the control group (*P* < 0.05). The E-selectin expression levels in the ipsilateral testes in the probucol-treated group were significantly lower than those in the ischemia-reperfusion group (*P* < 0.05). No significant difference was seen in the E-selectin expression level in the contralateral testes among the three groups (*P* > 0.05).

### 3.2. Myeloperoxidase and Malondialdehyde Results

**Figure 2:**
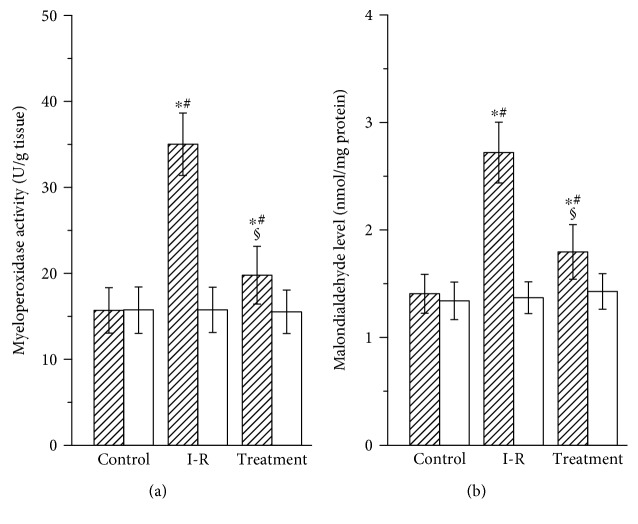
Myeloperoxidase activity (a) and malondialdehyde level (b) in ipsilateral (hatched bars) and contralateral (open bars) testicular tissues in the control, ischemia-reperfusion (I-R), and treatment groups. Results are expressed as mean ± standard deviation; *n* = 10. ^∗^*P* < 0.05 versus the control group; ^#^*P* < 0.05 versus contralateral testes in the same group; ^§^*P* < 0.05 versus ipsilateral testes in the I-R group.

All comparisons of testicular myeloperoxidase and malondialdehyde values in the three groups are shown in Figure 2. The myeloperoxidase and malondialdehyde values of the ipsilateral testes in the ischemia-reperfusion group were significantly higher than those in the control group (*P* < 0.05). The probucol-treated group had lower myeloperoxidase and malondialdehyde values in the ipsilateral testes than the ischemia-reperfusion group (*P* < 0.05). There were no significant differences among the three groups in the myeloperoxidase and malondialdehyde values of the contralateral testes (*P* > 0.05).

### 3.3. Evaluation of Testicular Spermatogenesis

**Figure 3:**
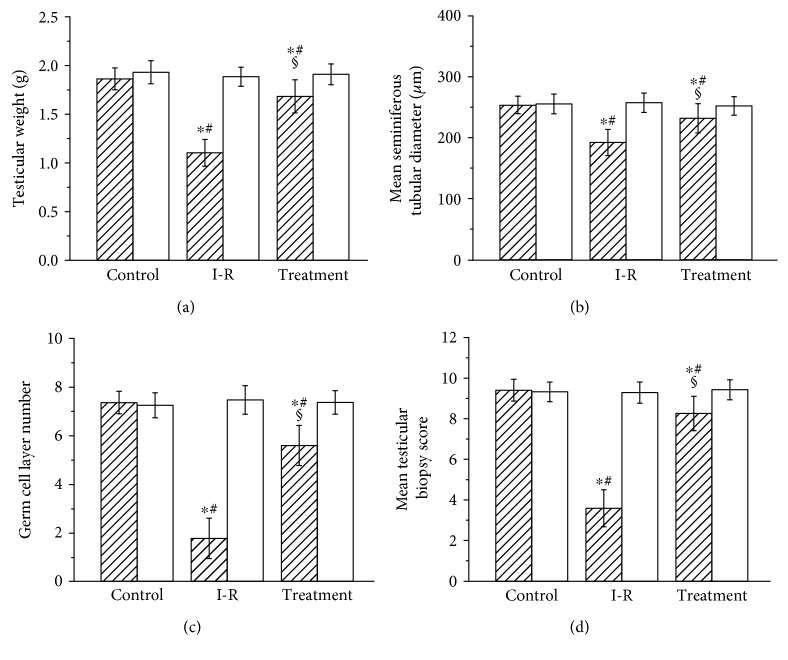
Testicular weight (a), mean seminiferous tubular diameter (b), germ cell layer number (c), and mean testicular biopsy score (d) in the ipsilateral (hatched bars) and contralateral (open bars) testes in the control, ischemia-reperfusion (I-R), and treatment groups. Results are expressed as mean ± standard deviation; *n* = 10. ^∗^*P* < 0.05 versus the control group; ^#^*P* < 0.05 versus contralateral testes in the same group; ^§^*P* < 0.05 versus ipsilateral testes in the I-R group.

**Figure 4:**
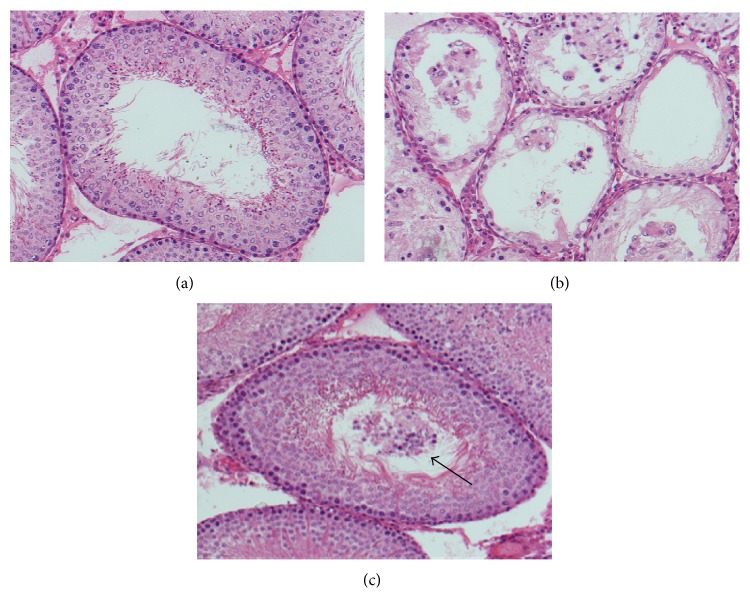
Microphotographs of cross sections of the seminiferous tubules in the control, ischemia-reperfusion, and treatment groups (hematoxylin and eosin staining, original magnification ×200). (a) Bilateral testes in the control group and contralateral testes in the ischemia-reperfusion and treatment groups all displayed normal seminiferous tubular diameter, germ cell layer number, and spermatogenesis from spermatogonium to spermatozoon. The germinal epithelium left an open lumen at the center of seminiferous tubule. (b) The ipsilateral testes in the ischemia-reperfusion group indicated atrophic seminiferous tubules, decreased germ cell layer number, and arrested spermatogenesis. (c) The ipsilateral testes in the treatment group showed nearly normal seminiferous tubule, but germinal epithelial cells sloughed into the tubular lumen (arrow), which easily occluded the seminiferous tubule.

Testicular weight, mean seminiferous tubular diameter, germ cell layer number, and mean testicular biopsy score in the three groups are presented in Figures 3 and 4. The values of the four parameters in the ipsilateral testes of the ischemia-reperfusion group were significantly lower than those of the control group (*P* < 0.05). The probucol-treated group showed significantly improved values of these parameters in the ipsilateral testes as compared with the ischemia-reperfusion group (*P* < 0.05). There were no significant differences in values of these parameters of the contralateral testes among the three groups (*P* > 0.05).

## 4. Discussion and Conclusions

Testicular torsion is an acute progressive disease, with an incidence of 1 in 4000 males by the age of 25 years [13]. Surgical detorsion should be performed promptly to avoid loss of testicular function. If testicular torsion is left untreated within 4–6 hours, necrosis of germ cells will occur [14]. Even if testicular torsion is corrected within this time period, testicular atrophy may develop subsequently [1]. In our study, rats underwent 2 hours of left testicular torsion followed by detorsion. Testicular torsion led to an ischemic purple appearance in the ipsilateral testis. After detorsion, the testis appeared normal in colour, suggesting that the testis is still viable. However, unilateral testicular torsion-detorsion produced pronounced injury in the ipsilateral testis 3 months after detorsion, including significant decreases in testicular weight, mean seminiferous tubular diameter, germ cell layer number, and mean testicular biopsy score.

The pathophysiologic mechanism of testicular damage is an ischemia-reperfusion injury. Testicular ischemia-reperfusion leads to overgeneration of reactive oxygen species [2]. Overproduced reactive oxygen species can damage lipids, proteins, and DNA, resulting in cellular dysfunction and even death [3]. It is very difficult to quantify reactive oxygen species directly because reactive oxygen species have high reactivity and short life span. Malondialdehyde is a stable end product of lipid peroxidation caused by reactive oxygen species [15, 16]. Therefore, it is generally accepted as a sensitive index of reactive oxygen species [15, 16]. In our study, unilateral testicular ischemia-reperfusion led to a significant increase in malondialdehyde level and a significant decrease in spermatogenesis in the ipsilateral testes, suggesting that overproduction of reactive oxygen species after testicular ischemia-reperfusion damages testicular spermatogenesis. Scavenging reactive oxygen species agents have been shown to be effective to protect against ischemia-reperfusion injury [17–19].

Probucol, a strong antioxidant, has been reported to protect against ischemia-reperfusion injury in the brain, heart, and kidney [8–10]. Furthermore, there is no adverse effect of probucol on male fertility [20]. Therefore, we attempted to use probucol for the treatment of testicular ischemia-reperfusion injury. Our study showed that probucol treatment significantly reduced malondialdehyde level and significantly improved spermatogenesis in the ipsilateral testes, compared with the ischemia-reperfusion group. These results suggest that probucol attenuates testicular injury by decreasing reactive oxygen species level. Probucol has been approved for clinical use [21]. It is safe and efficacious in the treatment and prevention of cardiovascular diseases [21]. As a result, we suggest that probucol treatment may have the clinical applicability in patients with testicular ischemia-reperfusion injury. However, the exact mechanism by which probucol reduces reactive oxygen species level in the testes still remains unknown.

One important source of reactive oxygen species production is neutrophils that infiltrate into the tissue itself [22]. E-selectin, an endothelial cell adhesion molecule, plays a key role in the recruitment of neutrophils to tissue [23]. Damage to vascular endothelium after ischemia-reperfusion of tissue leads to upregulation of E-selectin expression on endothelial cells [24–26]. Subsequently, E-selectin mediates neutrophil adhesion to vascular endothelium [27]. Finally, neutrophils transmigrate through vascular endothelium into tissue and release reactive oxygen species that cause tissular injury [27]. Myeloperoxidase is found predominantly in the granules of neutrophils [28]. Therefore, the enzyme activity is used as an indicator of neutrophil accumulation in tissue [28]. In our study, unilateral testicular ischemia-reperfusion caused significant increases in E-selectin expression, myeloperoxidase activity, and malondialdehyde level and caused a significant reduction in spermatogenesis in the ipsilateral testes. These results suggest that upregulation of E-selectin expression on vascular endothelial cells after testicular ischemia-reperfusion increases neutrophil accumulation in the testes and increased neutrophils produce excess reactive oxygen species, which can lead to spermatogenic injury in the ipsilateral testes. In addition, the present study showed that probucol treatment significantly decreased E-selectin expression, myeloperoxidase activity, and malondialdehyde level and significantly increased spermatogenesis in the ipsilateral testes. These imply that probucol treatment downregulates E-selectin expression on vascular endothelial cells and reduces neutrophil accumulation in the testes, resulting in a decrease in reactive oxygen species generation and an increase in spermatogenesis.

We have reported that curcumin [5] and rutin [6] can reduce testicular ischemia-reperfusion injury. The curcumin [5], rutin [6], and probucol protect the testes by different mechanisms. The curcumin exerts a protective effect on testicular ischemia-reperfusion injury by reducing reactive oxygen species formation by inhibiting xanthine oxidase [5]. The protective effect of rutin is caused by scavenging reactive oxygen species by increasing superoxide dismutase and catalase activities [6]. The probucol protects the testes from ischemia-reperfusion injury by reducing overgeneration of reactive oxygen species by inhibiting E-selectin protein expression and neutrophil accumulation in the testis. It is reasonable to speculate that the combination treatment with curcumin, rutin, and probucol may cause a much greater reduction in reactive oxygen species content and a much greater enhancement in the protective effect than monotherapy. Further studies are needed to confirm this hypothesis.

In the present study, therapy with probucol (300 mg/kg) significantly improved testicular spermatogenesis in the ipsilateral testes, but the saved spermatogenesis was still lower than normal value. A higher or lower dose of probucol may affect the effect of probucol on testicular spermatogenesis. Consequently, further studies will be needed to clarify the question so that probucol can achieve optimal effect.

There are conflicting results in the literature about whether unilateral testicular ischemia-reperfusion causes contralateral testicular damage. Some experimental studies showed that unilateral testicular ischemia-reperfusion resulted in contralateral testicular lesion [29, 30], while others reported no injury to the contralateral testis [31, 32]. In our study, we found that unilateral testicular ischemia-reperfusion caused significant changes in E-selectin protein expression, myeloperoxidase activity, malondialdehyde level, and spermatogenesis in the ipsilateral testis, but the contralateral testis did not show obvious changes in these parameters. Therefore, we believe that unilateral testicular ischemia-reperfusion has no effects on the contralateral testis.

Our study shows, for the first time, that probucol can attenuate testicular torsion/detorsion-induced ischemia/reperfusion injury by reducing overgeneration of reactive oxygen species by inhibiting E-selectin protein expression and neutrophil accumulation in the testis. It is worthy to be considered a pharmacological agent in clinical practice to treat patients suffering from testicular ischemia-reperfusion injury. For this purpose, further clinical investigations will be needed to determine whether probucol has clinical efficacy.
